# Supplementary material for: MetaRibo-Seq measures translation in microbiomes
Source: Nat Commun. 2020 Jun 29;11:3268. doi: 10.1038/s41467-020-17081-z (PMC7324362; doi:10.1038/s41467-020-17081-z)
Supplement: Supplementary file 10 — Supplementary Data 7 [file 41467_2020_17081_MOESM10_ESM.zip › File2/Confidence_High_Taxonomy/240764_out.krona.html]

Javascript must be enabled to view this page.

members
magnitude
magnitudeUnassigned
count
unassigned
taxon
rank

240764\_out

151

superkingdom
151
2

phylum
151
1239

class
186801
151

151
186802
order

541000
151
family

151
genus
1263
1

SRS1041145\_contig\_number\_contig-100\_18526.52491

2293165
12

SRS018541\_contig\_number\_4009SRS018817\_contig\_number\_22667SRS022524\_contig\_number\_7897SRS050925\_contig\_number\_21243SRS075716\_contig\_number\_307SRS098644\_contig\_number\_38423SRS140513\_contig\_number\_4991SRS150029\_contig\_number\_3895SRS893170\_contig\_number\_10680SRS893292\_contig\_number\_contig-100\_134.48557SRS893295\_contig\_number\_contig-100\_693.74338SRS893300\_contig\_number\_1045
species

species

SRS011084\_contig\_number\_36762SRS043701\_contig\_number\_contig-100\_26845.70316
2293161
2

165186
11

SRS011239\_contig\_number\_27469SRS011405\_contig\_number\_9302SRS015782\_contig\_number\_contig-100\_1021.235528SRS017191\_contig\_number\_518SRS024009\_contig\_number\_contig-100\_801.86424SRS045739\_contig\_number\_7972SRS1041038\_contig\_number\_8766SRS144297\_contig\_number\_12855SRS893279\_contig\_number\_14341SRS893373\_contig\_number\_contig-100\_1158.140257SRS971275\_contig\_number\_37679
species

14
2293149
species

SRS011302\_contig\_number\_18448SRS013638\_contig\_number\_18229SRS013965\_contig\_number\_27509SRS015431\_contig\_number\_contig-100\_982.474573SRS019068\_contig\_number\_103764SRS019808\_contig\_number\_contig-100\_899.112834SRS020394\_contig\_number\_13886SRS020869\_contig\_number\_contig-100\_588.225538SRS053335\_contig\_number\_9515SRS1041157\_contig\_number\_232SRS104912\_contig\_number\_contig-100\_923.117366SRS1055067\_contig\_number\_16159SRS140492\_contig\_number\_17223SRS147766\_contig\_number\_22248

species

SRS013940\_contig\_number\_20067SRS047044\_contig\_number\_contig-100\_31996.31996
2293156
2

species

SRS017103\_contig\_number\_contig-100\_370.231241SRS098061\_contig\_number\_contig-100\_612.90459SRS1055049\_contig\_number\_contig-100\_590.88278SRS143372\_contig\_number\_11069
2293233
4

15
1160721

SRS015486\_contig\_number\_contig-100\_557.558SRS018427\_contig\_number\_contig-100\_473.186637SRS022071\_contig\_number\_contig-100\_181.224785SRS023914\_contig\_number\_2794SRS024492\_contig\_number\_765SRS046717\_contig\_number\_6301SRS054352\_contig\_number\_22820SRS077194\_contig\_number\_2603SRS104084\_contig\_number\_13172SRS142980\_contig\_number\_contig-100\_559.52562SRS143780\_contig\_number\_8981SRS144362\_contig\_number\_12766SRS149075\_contig\_number\_626SRS893270\_contig\_number\_20014SRS971276\_contig\_number\_15766
species

3
1262962
species

SRS074670\_contig\_number\_6747SRS1055056\_contig\_number\_5021SRS893341\_contig\_number\_contig-100\_1998.39131

2293177
7
species

SRS014313\_contig\_number\_contig-100\_551.47767SRS019030\_contig\_number\_11286SRS064276\_contig\_number\_contig-100\_672.217516SRS076804\_contig\_number\_3136SRS077335\_contig\_number\_3617SRS104311\_contig\_number\_contig-100\_793.263585SRS147919\_contig\_number\_20794

2293178
2

SRS042628\_contig\_number\_35255SRS893369\_contig\_number\_8635
species

2293221
3
species

SRS019161\_contig\_number\_contig-100\_6117.216496SRS024663\_contig\_number\_13188SRS077641\_contig\_number\_contig-100\_2533.123764

species

SRS014923\_contig\_number\_38859SRS015663\_contig\_number\_15501SRS048164\_contig\_number\_19896SRS101433\_contig\_number\_contig-100\_599.101609SRS148319\_contig\_number\_16956SRS149181\_contig\_number\_5233
2293194
6

species

SRS015578\_contig\_number\_34716SRS104400\_contig\_number\_24733
1637508
2

species

SRS013476\_contig\_number\_contig-100\_1191.217399SRS014235\_contig\_number\_contig-100\_576.262156SRS014855\_contig\_number\_3423SRS014979\_contig\_number\_contig-100\_1101.190764SRS015579\_contig\_number\_26895SRS015694\_contig\_number\_23513SRS016018\_contig\_number\_7959SRS016629\_contig\_number\_2602SRS016954\_contig\_number\_23785SRS017821\_contig\_number\_15046SRS018133\_contig\_number\_contig-100\_2312.68211SRS018313\_contig\_number\_11385SRS018351\_contig\_number\_contig-100\_1240.105508SRS019178\_contig\_number\_80SRS019286\_contig\_number\_3774SRS019685\_contig\_number\_37601SRS019693\_contig\_number\_contig-100\_631.69232SRS019910\_contig\_number\_6549SRS019968\_contig\_number\_4987SRS024075\_contig\_number\_contig-100\_749.98688SRS024331\_contig\_number\_33469SRS043768\_contig\_number\_766SRS045195\_contig\_number\_contig-100\_940.63437SRS045826\_contig\_number\_1369SRS047014\_contig\_number\_17509SRS048870\_contig\_number\_21946SRS049446\_contig\_number\_contig-100\_556.90618SRS049712\_contig\_number\_21470SRS049773\_contig\_number\_contig-100\_692.198606SRS050752\_contig\_number\_13123SRS050941\_contig\_number\_21371SRS050998\_contig\_number\_3097SRS051882\_contig\_number\_contig-100\_407.129569SRS052027\_contig\_number\_contig-100\_589.119436SRS062427\_contig\_number\_contig-100\_1125.41106SRS063040\_contig\_number\_46625SRS063985\_contig\_number\_24378SRS065504\_contig\_number\_contig-100\_294.226513SRS075878\_contig\_number\_24315SRS077294\_contig\_number\_contig-100\_518.124403SRS097889\_contig\_number\_13996SRS098073\_contig\_number\_444SRS104636\_contig\_number\_contig-100\_262.149133SRS1055083\_contig\_number\_contig-100\_152.85614SRS142599\_contig\_number\_30082SRS142781\_contig\_number\_9196SRS142890\_contig\_number\_contig-100\_925.239723SRS143523\_contig\_number\_18213SRS144183\_contig\_number\_38701SRS147377\_contig\_number\_contig-100\_422.72179SRS147425\_contig\_number\_10833SRS147614\_contig\_number\_1207SRS148159\_contig\_number\_contig-100\_1153.312242SRS148874\_contig\_number\_5753SRS149325\_contig\_number\_contig-100\_681.96034SRS893259\_contig\_number\_5250SRS893288\_contig\_number\_3887SRS893358\_contig\_number\_contig-100\_487.77434SRS893378\_contig\_number\_17637
59
41978

species

SRS016989\_contig\_number\_contig-100\_447.101791SRS051031\_contig\_number\_35493
2293157
2


SRS012273\_contig\_number\_46580SRS016495\_contig\_number\_17037SRS054905\_contig\_number\_contig-100\_900.143449SRS144714\_contig\_number\_12850SRS147139\_contig\_number\_11906SRS148424\_contig\_number\_contig-100\_885.175655
species
2293169
6
